# ANKRD29, as a new prognostic and immunological biomarker of non–small cell lung cancer, inhibits cell growth and migration by regulating MAPK signaling pathway

**DOI:** 10.1186/s13062-023-00385-7

**Published:** 2023-06-06

**Authors:** Hanqing Zhao, Yanbo Wang, Yaomei He, Peng Zhang, Cheng Zeng, Tongxuan Du, Qiushuo Shen, Song Zhao

**Affiliations:** 1 Department of Thoracic Surgery, the First Affiliated Hospital of Zhengzhou University, 450052 Zhengzhou, China; 2 Academy of Medical Science, Zhengzhou University, 450052 Zhengzhou, China; 3 Institute of Biomedical Engineering, Kunming Medical University, 650500 Kunming, Yunnan China; 4 Yan’an Hospital Affiliated to Kunming Medical University, Kunming, China; 5 Key Laboratory of Tumor Immunological Prevention and Treatment in Yunnan Province, Kunming, China; 6 Center for Life Sciences, School of Life Sciences, Yunnan University, Kunming, Yunnan China

**Keywords:** ANKRD29, Non–small cell lung cancer, Cell growth and migration, Immune therapy response, Biomarker

## Abstract

**Background:**

The predominant cancer-related deaths worldwide are caused by lung cancer, particularly non-small cell lung cancer (NSCLC), despite the fact that numerous therapeutic initiatives have been devised to improve the outcomes. Ankyrin repeat domain (ANKRD) is one of the widespread protein structural motifs in eukaryotes but the functions of ANKRD proteins in NSCLC progression remains unclear.

**Methods:**

We performed integrative bioinformatical analysis to determine the dysregulated expression of ANKRDs in multiple tumors and the association between ANKRD29 expression and the NSCLC tumor environment. Quantitative real-time PCR (qRT-PCR), western blot, immunohistochemistry (IHC), and tissue microarray (TMA) assays were used to investigate the expression of ANKRD29 in NSCLC cell lines. The role of ANKRD29 in NSCLC cell proliferation and migration in vitro was deteceted by 5-bromodeoxyuridine (BrdU) incorporation, colony formation, flow cytometry, would-healing, trans-well, and western blot experiment. RNA-seq technology was applied to deciper the molecular mechanism regulated by ANKRD29 in NSCLC.

**Results:**

We constructed a valuable risk-score system for predicting the overall survival outcomes of NSCLC patients based on the expression of five hub ANKRD genes. And we found that the hub gene ANKRD29 was remarkedly decreased in NSCLC tissues and cell lines due to the promoter hypermethylation, and revealed that high ANKRD29 expression obviously correlated with patients’ better clinical outcome. Overexpression of ANKRD29 significantly inhibited cell proliferation and migration, promoted the cancerous cells’ sensitivity to carboplatin and enhanced the killing ability of T cells in NSCLC cells. Interestingly, ANKRD29 can be served as a biomarker to predict the response to immunotherapy in NSCLC. Mechanically, RNA-seq results showed that ANKRD29 could regulate MAPK signaling pathway. Moreover, we screened two potential agonists for ANKRD29.

**Conclusions:**

ANKRD29 functions as a new tumor suppressor in NSCLC tumorigenesis and could be developed as a biomarker for prognostic prediction, immunotherapy response, and drug susceptibility evaluation of NSCLC in the future.

**Supplementary Information:**

The online version contains supplementary material available at 10.1186/s13062-023-00385-7.

## Introduction

Lung cancer is one of the dominant malignancies and the leading cause of cancer-related deaths worldwide among men and women, which accounting for approximately one-tenth (11.4%) of all diagnosed cancers and one-fifth (18.0%) of all cancer deaths [1, 2]. More than 85% of patients were diagnosed with non-small cell lung cancer (NSCLC), of which lung adenocarcinoma (LUAD) and lung squamous carcinoma (LUSC) were the most common histologic subtypes [3]. Although ever-evolving strategies for early diagnosis and currently available treatment options, including surgery, radiotherapy, chemotherapy (such as carboplatin), molecularly targeted therapy, and immunotherapy, have significantly improved the therapeutic outcomes, the NSCLC prognosis is still poor, with a poor 5-year survival of about 15% [2]. Even worse, many NSCLC patients were diagnosed with distant metastasis, which severely augment the difficulty of treatment and decrease patient survival rates [4]. Therefore, it is essential to develop new prognostic biomarkers and explore the molecular mechanisms of NSCLC progression.

Ankyrin repeat domain (ANKRD) is one of the widespread protein structural motifs in eukaryotes [5]. It was first identified as the cell cycle regulators (Swi6 and Cdc10) of Saccharomyces cerevisiae, and this module containing 24 copies was subsequently named cytoskeletal protein ankyrin protein [6]. A typical ANKRD usually contains 33–34 amino acid residues and rely on the interfolding of the structural regions to form a β-hairpin–α-helix–loop–α-helix (β2α2) structure. Some ANKRD proteins consist of only ANK repeats, while others are multidomain molecules in which ANK repeats are combined with other unrelated modules [7, 8]. ANKRD has multiple functions, such as transcription initiation, cell cycle regulation, developmental regulation, cytoskeleton maintenance, and intercellular signaling [5, 9]. Recently, several studies have demonstrated that ANKRD proteins could regulate essential cellular processes including proliferation, migration, apoptosis, epithelial mesenchymal transition (EMT), and drug sensitivity [10–16]. For example, ANKRD22 activated the Wnt/β-catenin signaling pathway by regulating the expression of NuSAP1 to promote breast cancer cells’ proliferation, invasion and EMT ability [12]. Additionally, down-regulation of ANKRD1 expression increased the sensitivity of ovarian cancer to cisplatin [14, 17]. Heretofore, we know little about the roles of other ANKRDs in tumors including NSCLC, such as ANKRD2 to ANKRD65.

In this study, we construct a prognosis risk model to predict the overall survival (OS) outcomes of NSCLC patients based on the expression of five hub ANKRD genes (ANKRD29, ANKRD34B, ANKRD40CL, ANKRD44 and ANKRD66). Moreover, we found five hub genes were dysregulated in NSCLC and overexpression of ANKRD29 inhibited NSCLC cells growth and migration process. RNA-seq results indicated ANKRD29 suppressed NSCLC malignance may through MAPK signaling pathway. And we revealed that ANKRD29 could be involved in tumor microenvironment (TME) and served as a promising biomarker of immune checkpoint blockers (ICBs) therapy in NSCLC by integrative bioinformatical analysis and T cell killing assays. Collectively, we proved that ANKRD29 played important roles in NSCLC progression.

## Materials and methods

### Datasets and data processing

Gene expression data and clinical information of LUAD and LUSC samples (108 normal and 1037 tumor samples) were downloaded from TCGA (https://portal.gdc.cancer.gov/). For GEO datasets, gene expression data were downloaded from NCBI GEO website and analyzed by GEO2R online software [18]. Considering the hundreds of ankyrin-like repeats in functionally diverse proteins, we focused on 75 ANKRDs (named directly as ANKRD, Table S1) for further studies according to previous literatures and the gene module of National Center for Biotechnology Information (NCBI) website. Other databases performed in this study were available to the public and details are shown in Table S7.

### Differential expression gene (DEGs) analysis

To identify the DEGs between tumor and normal NSCLC samples, TCGA datasets were analyzed by the “DESeq2” package of R software (version 3.6.3) [19]. The DEGs were filtered using a threshold of adjusted P < 0.05 and an absolute log2-fold change > 1. DEGs were visualized by plotting volcanoes using the “ggplot2” packages (version 3.3.3) [20]. In GEO datasets (GSE118370, GSE19804, GSE33532, GSE18842 and GSE31548), the DEGs were identified by top 150 significantly down-regulated genes in tumors compared to normal tissues, and Venn analysis was used to select overlapping DEGs among the five algorithms mentioned above.

### Constructing the risk-score system

Venn analysis was used to select overlapping genes between DEGs of TCGA datasets and 75 ANKRDs mentioned above. And Univariate Cox proportional hazards regression was performed with the DEGs selected for the risk-score system (P < 0.05). To narrow down the overfitting high-dimensional prognostic genes, we performed the Least Absolute Shrinkage and Selection Operator (LASSO)-regression model assay by “glmnet” package [21]. Next, Multi-factor cox regression analysis was performed to obtain the regression coefficients of independent prognostic factors β. The Risk Score System (RSS) was then established. The formula is as following: RSS = EXP (gene 1) ×β1 + EXP (gene 2) ×β2 + EXP (gene 3) ×β3+. . + EXP (gene n) ×βn, in which EXP denotes the expression level of risk factor [22]. Patients were divided into low- and high-risk groups by calculating the Risk Score for each TCGA-NSCLC sample using the median as the threshold value, and OS was compared between the two groups by Kaplan-Meier (KM) curves. The receiver operating characteristic (ROC) curve was used for analyzing the prognostic predictive value of RSS in patients with NSCLC.

### Development and evaluation of nomogram

To comprehensively assess patient survival, we performed univariate and multivariate Cox regression analyses of clinicopathological parameters, including age, TNM stages, and risk group. We used the “rms” package to construct a Nomogram integrating these parameters to assess patient survival at 1-, 3-, and 5 years. The calibration curves were used to assess the suitability of our constructed Nomogram for clinical application [23].

### Methylation analysis and 5-Aza-2’-Deoxycytidine (5-Aza) treatment

cBioPortal (https://www.cbioportal.org/) was applied to detect mutation status and the correlation between ANKRD29 expression and promoter methylation level [24, 25]. The SMART and MethSurv web tools were performed to visualize ANKRD29 expression and promoter (CpG islands) methylation levels, and evaluate the prognosis of ANKRD29 methylation levels [26, 27]. In 5-Azacytidine (5-Aza) treatment experiment, indicated cells were treated with 5 µM 5-Aza (Selleck) for 24 h and collected for RNA and protein extraction for further qRT-PCR and immunoblot experiments.

### Immune infiltration analysis

The TIMER database (https://cistrome.shinyapps.io/timer/) was used to analyze the correlation between copy number alternation of ANKRD29 and immune cell infiltration levels, including B cells, CD4 + T cells, CD8 + T cells, Neutrophils, Macrophages, and dendritic cells [28]. Single-sample gene set enrichment analysis (ssGSEA) algorithm, based on the “GSVA” R package (version 1.34.0), was used to calculate the enrichment scores of immune cell types in tumor microenvironment to determine the immune cell infiltration level in NSCLC [29]. The ESTIMATE algorithm was used to calculate the stromal scores, immune scores and the ESTIMATE score (based on the “ESTIMATE” R package, version 1.0.13) [30]. The TISCH datasets (http://tisch.comp-genomics.org/) was used to investigate the expression profile of ANKRD29 in NSCLC tumor microenvironment [31]. For immunophenoscore (IPS) assay, the correlation between ANKRD29 expression and IPS score were analyzed by “IOBR” R package (version 0.99.9) in pan-cancer [32]. The full names of pan-cancer were shown in Table S9.

### Constructs and cell culture

The human coding DNA sequence of ANKRD29 was synthesized (Tsingke Biotechnology Co., Ltd.) and cloned into pCDH-MSCV-E2F-eGFP lentiviral vector using NheI and EcoRI restriction endonuclease sites. Lenti-viruses were generated according to the manufacture protocol, cells were infected by viruses twice with 48 and 72 h viral supernatants containing 4 µg/mL polybrene for further stable overexpressing cells screening by puromycin. The pCDH-MSCV-E2F-eGFP vector was used as an empty vector control. HKE-293T was obtained from ATCC. 16HBE, A549, H1299, SPC-A1, H1650 and H1975 were purchased from Cell Bank of Kunming Institute of Zoology with STR document. HKE-293T cells were cultured in DMEM medium (Gibco). 16HBE, A549, H1299, SPC-A1, H1650 and H1975 cells were cultured in RPMI1640 medium supplemented with 10% fetal bovine serum (FBS) and 1% penicillin/streptomycin. All cells were cultured 37℃, 5% CO_2_ humidified environment.

### Cell proliferation, BrdU incorporation and colony formation assays

To determine the growth ability of cells, indicated NSCLC cells were plated into 12-well plates for 1 × 10^4^ cells/well and the cell numbers were subsequently counted each day using an automatic cell analyzer Countstar (Shanghai Ruiyu Biotech Co., China, IC 1000). For BrdU incorporation, cells were seeded into 8-well plate for 24 h culture. Cells were treated with 10µM BrdU (Abcam) for 20 min and then fixed with 5% PFA. After permeabilization, cells were incubated with BrdU (Cell Signaling Technology) primary antibody overnight and secondary antibody (Thermo). The nucleus was stained with DAPI. In the colony formation assay, indicated cells were seeded into 6-well plate with 400 cells/well, and the medium was changed every 3 days for 2 weeks. After which, cells were fixed with 5% PFA and stained with crystal violet for images capture and counting.

### Wound-healing and trans-well assays

For wound healing assay, stable ANKRD29 overexpressing and control cells were plated into 6-well plate at 1 × 10^6^ cells/well. After 24 h, the adherent cell monolayer was scratched by an Eppendorf tip. PBS was used to remove of floating cells. Wounds were imaged at indicated time points and measured by image J software. For trans-well assay, cells in serum-free medium were seeded onto chemotaxis chambers (Corning). Medium with 10% FBS was added to the bottom wells of chambers. Cells were cultured in 37 °C for 24 h. The non-migrated cells in the upper chamber were removed cautiously by using a cotton swab, and the migrated cells adhered to the lower membrane were stained with crystal violet. Migrated cells were measured with an inverted microscope.

### Flow cytometry analysis and sulforhodamine B (SRB) staining

To detect the cycle cell population distribution affected by ANKRD29 overexpression, indicated cells were pre-treated with 12 h starvation and then cultured with complete medium for another 12 h. Cells digested with trypsin were fixed in 75% ethanol for overnight and stained by propidium iodide for 30 min at 37 °C, and then analyzed by flow cytometer. To detect the sensitivity of tumor cells for carboplatin treatment, SRB assay was applied. Briefly, cells were seeded into 96-well plate with 1 × 10^4^ cells/well. After 24 hours’ culture, the medium containing the corresponding concentration of the drug was removed and changed. Cells were fixed with trichloroacetic acid (50%), washed and stained with 0.4% SRB. OD values were measured at 510 nm using a microplate reader.

### Quantitative real Time-PCR (qRT-PCR) and western blot (WB)

Total RNA was extracted from cells using RNAiso Plus (Takara) according to the instructions. Reverse transcription was performed using the PrimeScript RT kit (Takara). The qRT-PCR was performed using FastStart Universal SYBR Green Master Mix (Roche). The qRT-PCR primers of human gene ANKRD29 and β-actin were shown in Table S8. For WB assays, cells were lysed with RIPA buffer on ice. Protein concentrations were measured and the supernatant was then boiled at 100 °C for 5 min. Protein samples were separated by SDS-PAGE electrophoresis and transferred to polyvinylidene fluoride membranes (Millipore). The samples were then blocked with 5% skim milk and incubated with different specific primary and secondary antibodies for further analysis by a MiniChemi imaging system. The antibodies used in this study are shown in Table S8.

### T cell-mediated cancer cell killing assay

To analyze the killing effect of T cells on tumor cells, we co-cultured NSCLC cells with activated human T cells (Jurkat) for 2 days as previous reported [33] in 12-well plates. After which, the wells were washed twice with PBS to remove the T cells. The surviving tumor cells were then fixed and stained with crystal violet solution. The dried plates were photographed and their intensity was quantified at OD_570_.

### Tissue microarrays and immunohistochemistry (IHC) assays

NSCLC tissue microarrays (TMA) with clinical and pathological information were purchased from Shanghai Zhuoli Biotechnology Co further IHC analysis. The sample size was 80 cases. Pathological grading, TNM staging, and clinical information were available for all patients. For immunohistochemistry, assay was performed as described previously [34]. This study was reviewed and approved by the Ethics Committee of the First Affiliated Hospital of Zhengzhou University (approval no. 2020-KY-277).

### Statistical analysis

Statistical differences between two groups were calculated by student’s t-test, comparisons between three or more groups were performed by one-way ANOVA, correlations were analyzed by Spearman’s correlation coefficient, and survival curves were expressed by KM plotter and log-rank tests. GraphPad Prism 8.4 (CA, USA) and R software (version 3.6.3) were used to process and analyze the data. P < 0.05 was considered statistically significant (* P < 0.05, ** P < 0.01, *** P < 0.001).

## Results

### Construction and assessment of the risk-score system (RSS) based on ANKRDs

**Fig. 1:**
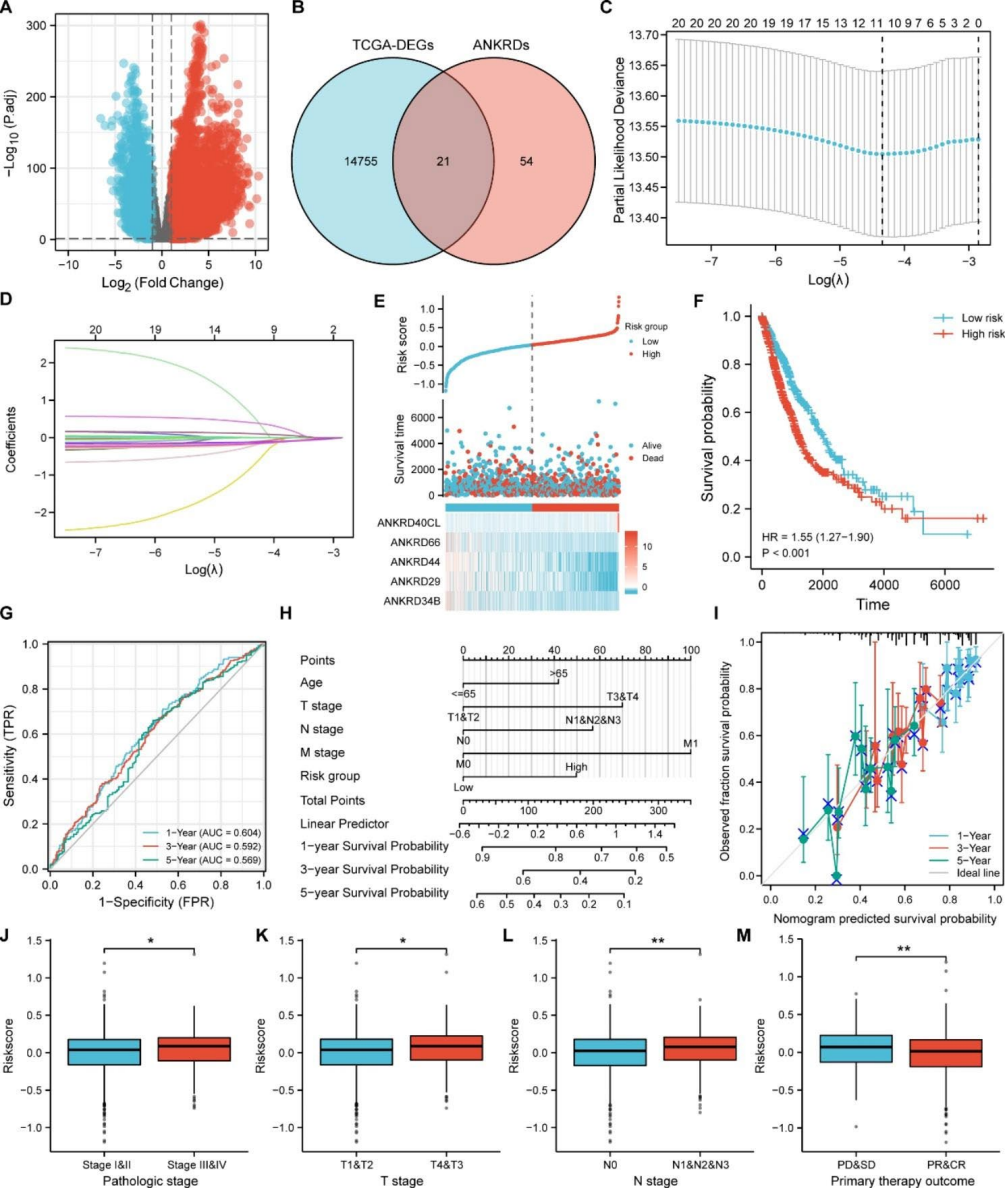
Establishment of risk-score system. **A** Volcano plot of differentially expressed genes (DEGs) using TCGA-NSCLC datasets. **B** The Venn diagram of the overlapping genes of DEGs and 75 ANKRD genes. **C** Cross-validation for parameter selection in the LASSO regression model. **D** Coefficient profiles for the LASSO model. **E** Risk score and survival time, and heatmap of the five ANKRD genes in the TCGA database. **F** Kaplan-Meier curves were performed to estimate the OS time in the high-risk and low-risk groups of NSCLC patients. **G** Time-dependent ROC curve for predicting 1-, 3-, and 5- year survival. **H** Nomogram for predicting 1-, 3-, and 5- year OS of NSCLC patients. **I** Calibration curve for the OS nomogram model in NSCLC patients. **J-M** Relationship between clinicopathological characteristics and risk scores based on the five ANKRD genes. The distribution of risk scores showed statistically significant differences in NSCLC patients stratified by WHO stage (**J**), T stage (**K**), N stage (**L**) and primary therapy outcome (**M**). ROC: receiver operating characteristic; OS: overall survival. Bars are the mean value ± SD. ns = no significant. * P < 0.05, ** P < 0.01, *** P < 0.001

To determine whether ANKRDs plays important role in NSCLC progression and develop new biomarkers for NSCLC prognosis based on ANKRDs expression, we selected 75 directly named ANKRDs for further analysis in view of the hundreds of ankyrin-like repeats in functionally diverse proteins (Table S1). Firstly, we performed the differential expression genes (DEGs) assay in NSCLC tumor samples compared to normal tissues. We obtained 14,776 DEGs significantly down/up-regulated in TCGA-NSCLC dataset and visualized them with volcano maps (Fig. 1A). Venn Diagram analysis between DEGs and ANKRDs indicated that 21 ANKRDs were dysregulated in NSCLC (Fig. 1B and Table S2). Next, univariate Cox regression was used to detect the correlation between the ANKRDs expression levels and overall survival (OS) time and we identified 5 hub risk factors (ANKRD29, ANKRD34B, ANKRD40CL, ANKRD44 and ANKRD66), which was confirmed by the LASSO regression algorithm analysis (Fig. 1C, D, and Table S3). Subsequently, we performed multi-factor cox regression analysis to obtain the regression coefficients of independent prognostic factors and established the risk-score system using the formula mentioned in Materials and Methods (Table S4). And the risk score for each patient in TCGA NSCLC datasets was calculated and shown (Fig. 1E). Moreover, NSCLC patients with high-risk score remarkedly exhibited worse overall survival time compared to those with low-risk score and receiver operating characteristic (ROC) curve analysis showed that the AUC values of patients at 1-, 3- and 5- year were 0.604, 0.592 and 0.569, respectively (Fig. 1F, G). Additionally, to verify the independence of RSS for predicting the OS time of NSCLC patients, the potential predictors (age, TNM stages and risk group) were analyzed by univariate Cox regression and were integrated into the nomogram model using TCGA-NSCLC datasets, and the results revealed that age, TNM stages and risk level were considered as independent risk factors for OS (Fig. 1H and Table S5). Besides, the calibration plots showed nice agreement between the 1-, 3-, and 5-year OS rates, when comparing the nomogram model and the ideal model (Fig. 1I). Importantly, the risk-scores were elevated in malignant stages and displayed worse sensitivity to primary therapy outcome of NSCLC patients (Fig. 1J-M). In summary, the RSS based on five hub ANKRD genes showed a significant association with survival of NSCLC.

### Five hub ANKRD genes were dysregulated in NSCLC

**Fig. 2:**
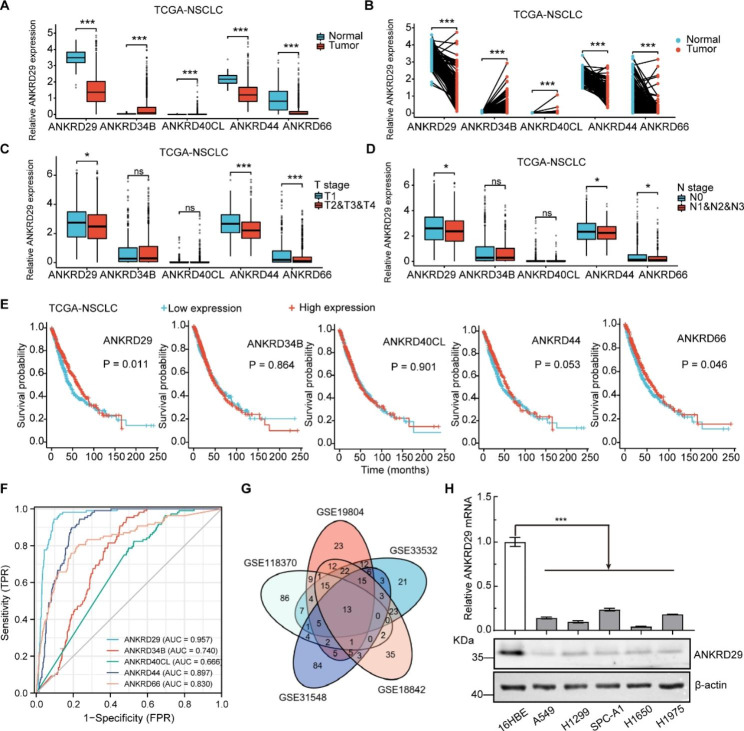
Five ANKRD genes were dysregulated in NSCLC. **A-B** Expression levels of the five ANKRD genes between NSCLC and normal tissues from TCGA cohorts. **C-D** Correlation analysis between five risk factors expression and stages of NSCLC were performed. **E** The Kaplan-Meier survival analysis of five ANKRDs expression in TCGA NSCLC dataset. **F** ROC curves of indicated genes in NSCLC from TCGA database. **G** Venn diagram analysis of 13 DEGs from five GEO datasets of NSCLC. The 13 DEGs were CLDN18, STXBP6, PTPRB, PDK4, BTNL9, ADRB1, EMCN, ABI3BP, GIMAP8, ROBO4, HHIP, ANKRD29 and ADAMTS8. **H** Relative mRNA and protein expression levels of ANKRD29 in NSCLC cell lines including A549, H1299, SPC-A1, H1975 and H1650. Normal human bronchial epithelial cells 16HBE as control. ROC: receiver operating characteristic; TCGA: The Cancer Genome Atlas. Bars are the mean value ± SD. ns = no significant. * P < 0.05, ** P < 0.01, *** P < 0.001

To validate the expression of five hub ANKRD genes in TCGA-NSCLC datasets, we found that ANKRD29, ANKRD44 and ANKRD66 were significantly downregulated, while the mRNA expression levels of ANKRD34B and ANKRD40CL were upregulated in NSCLC tumor tissues compared to normal tissues in both paired and unpaired samples (Fig. 2A, B). And the expression of ANKRD29, ANKRD44 and ANKRD66 were decreased in advanced stages of NSCLC, but ANKRD34B and ANKRD40CL showed no difference (Fig. 2C, D). In addition, Kaplan-Meier survival curves proved that low expression levels of ANKRD29, ANKRD44 and ANKRD66 were associated with shorter overall survival compared to those with high expression levels in NSCLC patients (Fig. 2E), suggesting ANKRD29 could be more essential for NSCLC progression among these five hub genes, which was confirmed by mutation assays in cBioPortal websites and ROC analysis of TCGA-NSCLC datasets (Fig. S1A, B). In addition, we clarified the among risk factors expression through correlation analysis and protein-protein interaction (PPI) network analysis in STRING database (https://string-db.org/) (Fig. S1C, D). Considering the three essential ANKRDs (ANKRD29, ANKRD44 and ANKRD66) were congruously down-regulated in NSCLC, we filtrated the most critical ANKRD in NSCLC development using GEO datasets (GSE118370, GSE19804, GSE33532, GSE18842 and GSE31548) and identified 13 candidate genes (CLDN18, STXBP6, PTPRB, PDK4, BTNL9, ADRB1, EMCN, ABI3BP, GIMAP8, ROBO4, HHIP, ANKRD29 and ADAMTS8), whose expression was obviously decreased in cancerous tissues compared to normal tissues (Fig. 2G). Therefore, we selected ANKRD29 for further study. To validate the lower expression of ANKRD29 in NSCLC cells, qRT-PCR and western blot results revealed that ANKRD29 expression was decreased in NSCLC cancerous cell lines (A549, H1299, SPC-A1, H1650 and H1975), in which the normal human bronchial epithelial cell lines (16HBE) was the control cell line (Fig. 2H).

### Promoter hypermethylation resulted in ANKRD29 low expression in NSCLC

**Fig. 3:**
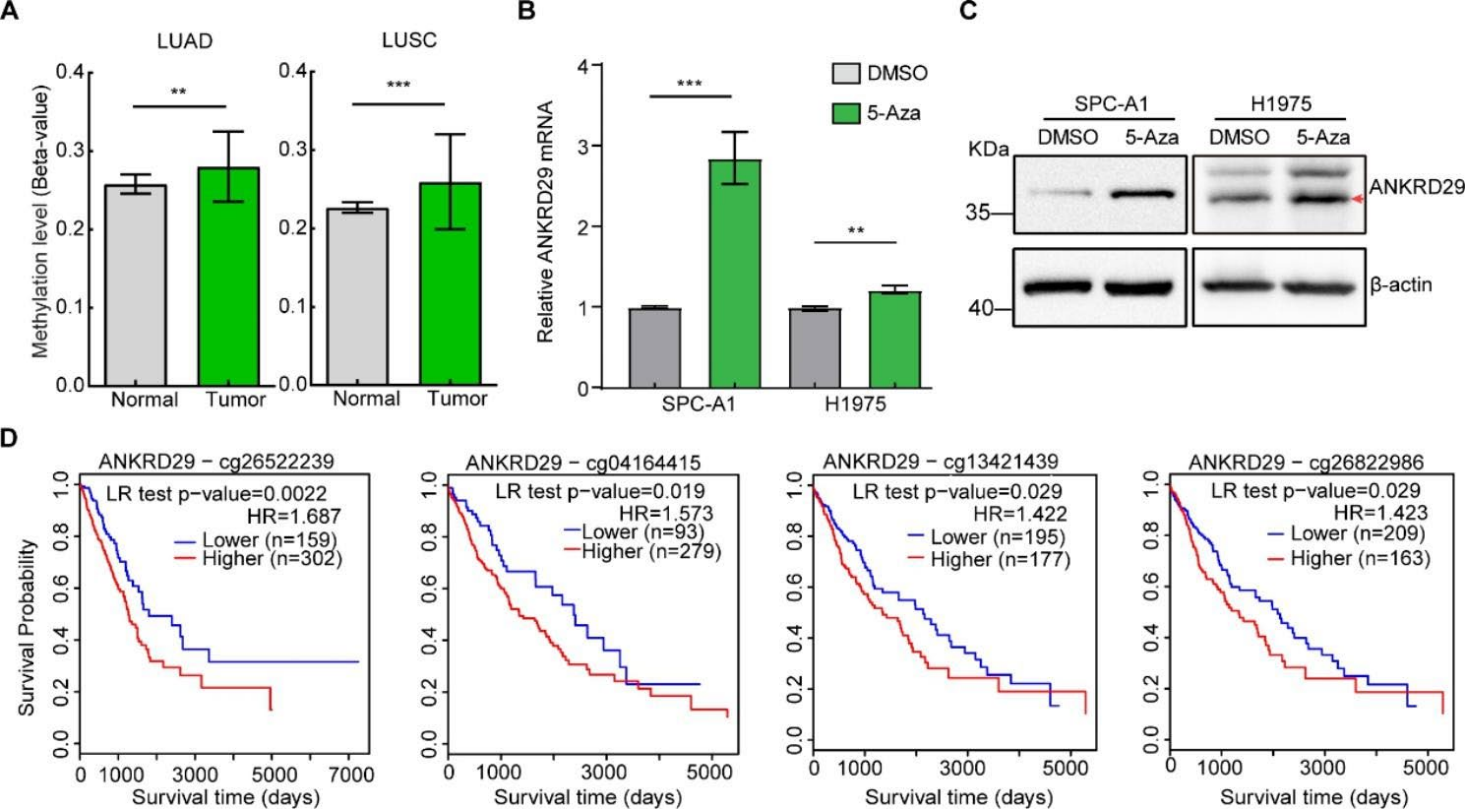
Promoter hypermethylation induced ANKRD29 low expression in NSCLC. **A** Methylation levels in normal tissues and LUAD or LUSC tissues from SMART website. **B-C** ANKRD29 expression was detected by qRT-PCR (**B**) and immunoblot (**C**) in NSCLC cells treated with 5-Azacytidine (5-Aza) or DMSO. The red arrow indicated the target band of ANKRD29 protein. **D** Four ANKRD29 CpG sites of high methylation levels including cg26522239, cg04164415, cg13421439 and cg26822986 predicted a poor prognosis. Bars are the mean value ± SD. ns = no significant. * P < 0.05, ** P < 0.01, *** P < 0.001

To clarify the underlying mechanism by which ANKRD29 was downregulated in NSCLC, we examined the correlativity between the methylation status of ANKRD29 promoter and expression in cBioPortal, SMART and MethSurv databases [25–27]. We found that the methylation levels of ANKRD29 promoter were significantly increased in tumors compared to normal tissues and ANKRD29 expressions were negatively correlated to promoter methylation levels in LUAD and LUSC (Fig. 3A; Fig. S2A, B). More importantly, ANKRD29 mRNA and protein expression levels were conspicuously elevated after treatment with DNA methyltransferase inhibitor 5-Aza in SPC-A1 and H1975 cells (Fig. 3B, C). Besides, the ANKRD29 expression was negatively associated with DNMT3B expression in LUAD and LUSC, suggesting DNMT3B could mediate the low expression of ANKRD29 in NSCLC (Fig. S2C). Additionally, the Kaplan-Meier survival curves analysis showed that high methylation levels at four CpG sites in the ANKRD29 gene were associated with poor prognosis, including cg26522239 in LUAD and cg04164415, cg13421439 and cg26822986 in LUSC (Fig. 3D). Collectively, we hypothesized that decreased expression of ANKRD29 was due to promoter hypermethylation and was critical for NSCLC tumorigenesis.

### Overexpression of ANKRD29 inhibited NSCLC cell proliferation and induced cell cycle arrest

**Fig. 4:**
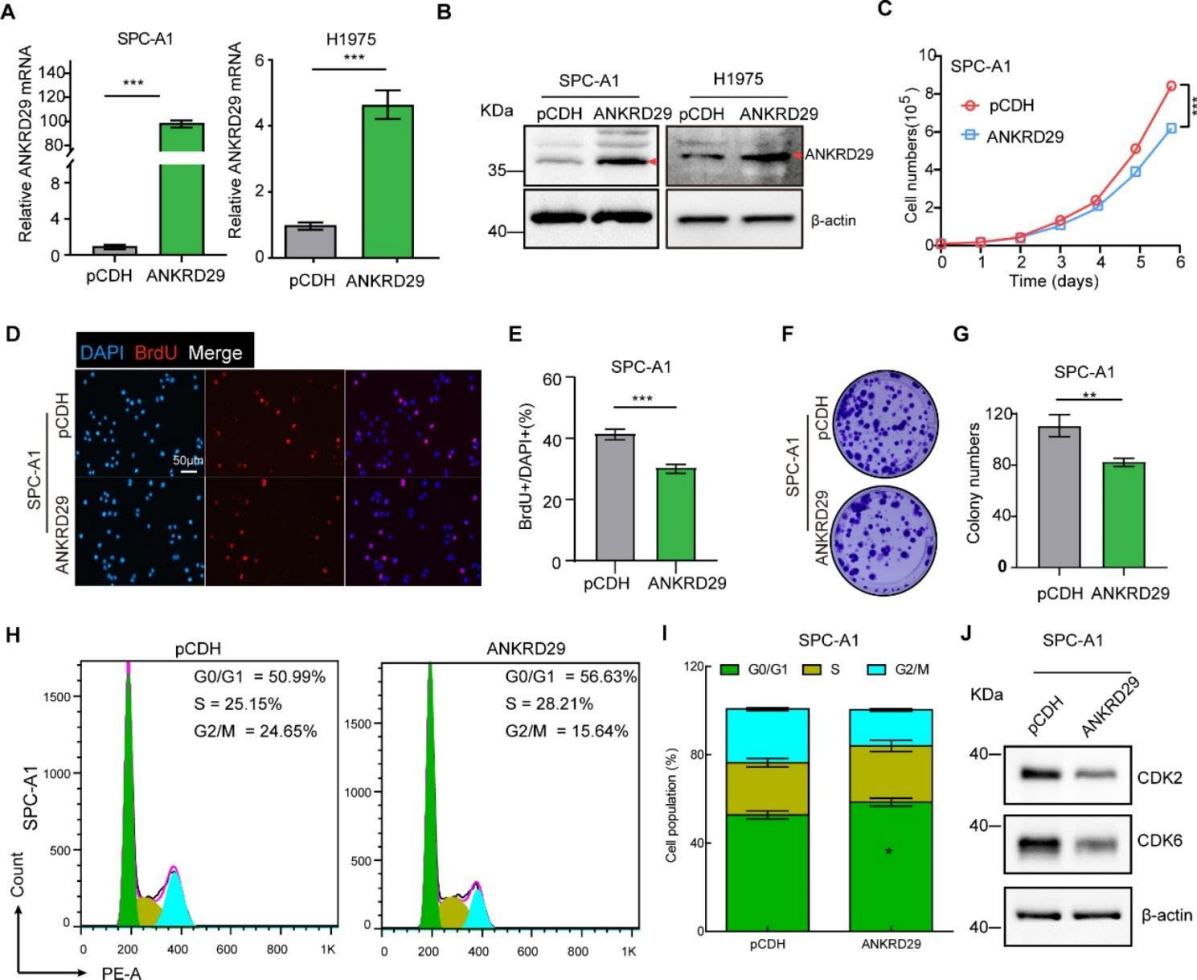
Overexpression of ANKRD29 inhibited cell proliferation and regulated cell cycle. **A-B** qRT-PCR (**A**) and western blot (**B**) assays were used to verify the overexpression of ANKRD29 in SPC-A1 and H1975 cell lines. The red arrow indicated the target band of ANKRD29 protein. **C-G** Overexpression of ANKRD29 obviously inhibited SPC-A1 cell growth through growth curve (**C**), BrdU incorporation abilities (**D-E**) and colony formation (**F-G**). (**E**) and (**G**) Quantification data for (**D**) and (**F**), respectively. **H-I** Overexpression of ANKRD29 induced cell cycle arrest at G0/G1 phase analyzed by flow cytometry assay. (**I**) Quantification data for (**H**). **J** ANKRD29 elevated expression downregulated the protein level of CDK2 and CDK6. Scale bar = 50 μm. Bars are the mean value ± SD. ns = no significant. * P < 0.05, ** P < 0.01, *** P < 0.001

To determine whether ANKRD29 regulate NSCLC cell growth, we first examined the effect of ANKRD29 silencing in NSCLC cells using the database of Novartis DRIVE cancer cell lines (https://oncologynibr.shinyapps.io/drive/), which providing the cancer community with gene dependence profiles across nearly 400 cell lines. [35], and found that knock-down of ANKRD29 by shRNA only promotes cell growth in a part of NSCLC cells (ATARiS > 0, Fig. S3A). Considering the low expression in NSCLC cells, we overexpressed ANKRD29 in SPC-A1 and H1975 cell lines, which was verified by qRT-PCR and western blot assays (Fig. 4A, B). As expected, overexpression of ANKRD29 inhibited the proliferation and reduced the proportion of BrdU-positive cells of SPC-A1 (Fig. 4C-E) and H1975 (Fig. S3C-E) in vitro compared to the respective control groups. In addition, we observed a significant abatement of colony formation ability in SPC-A1 and H1975 cells by ANKRD29 upregulation (Fig. 4F, G; Fig. S3F, G). We then analyzed the cell cycle transition by flow cytometry and found that overexpression of ANKRD29 increased the proportion of G0/G1 phase cell populations in both cell lines (Fig. 4H, I; Fig. S3H, I). Consistently, the protein levels of CDK2 and CDK6 were decreased in the ANKRD29 overexpression group compared to the control group, which were two key G0/G1 checkpoints (Fig. 4J; Fig. S3J). Taken together, ANKRD29 overexpression suppressed the growth ability of NSCLC cells and induced cell cycle arrested at G0/G1 phase.

### Elevated ANKRD29 expression restrained NSCLC cell migration

**Fig. 5:**
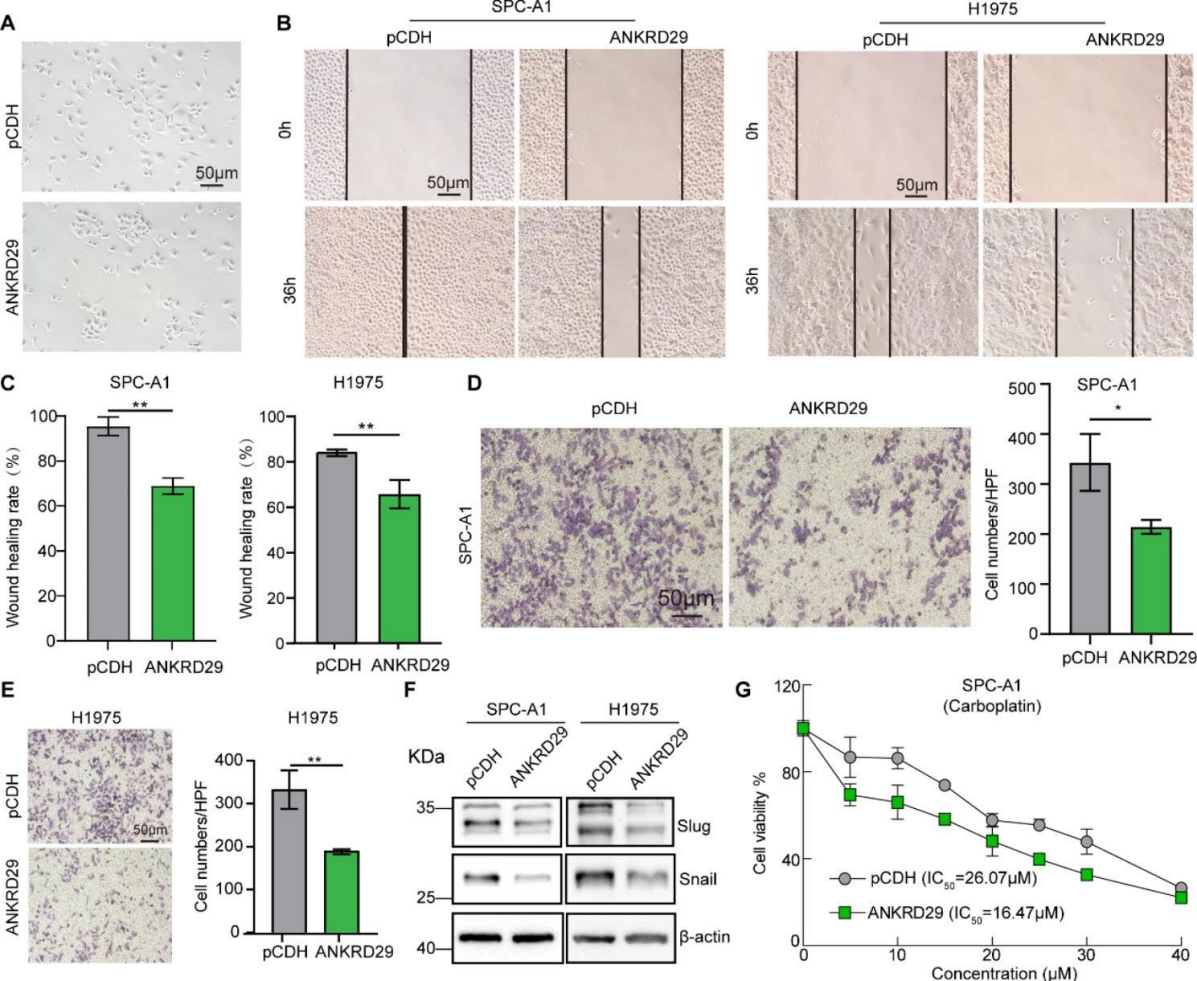
Overexpression of ANKRD29 repressed NSCLC cell migration. **A** Representative photograph demonstrating the morphological change of H1975 after ANKRD29 overexpression in bright field. **B-C** SPC-A1 and H1975 cells’ migration abilities were inhibited after the overexpression of ANKRD29 by wound-healing assays. (**C**) Quantification data for (**B**). **D-E** ANKRD29 Overexpression suppressed SPC-A1 and H1975 cells’ migration abilities by trans-well assays. **F** The expressions level of EMT-related protein Slug and Snail were determined by Western blotting. **G** IC_50_ values were determined by SRB assays in ANKRD29 overexpressing cell and control group of SPC-A1. Scale bar = 50 μm. Bars are the mean value ± SD. ns = no significant. * P < 0.05, ** P < 0.01, *** P < 0.001

To explore the regulation of ANKRD29 on the migration of NSCLC cells, we firstly examined the morphological changes in H1975 cells after forced-expression of ANKRD29. We found that the control group cells presented a typical mesenchymal-like morphology, while the overexpression of ANKRD29 changed the cells to display an epithelial-like morphology (Fig. 5A). Subsequently, to demonstrate the regulation of cell migration by ANKRD29, we performed wound healing assays and trans-well assays. The results showed that the migration abilities were markedly suppressed in the ANKRD29 overexpressing groups compared to control groups (Fig. 5B-E). Immunoblotting results of Slug and Snail reduction in ANKRD29 overexpressed SCP-A1 and H1975 cells were consistent with this conclusion (Fig. 5F). In addition, recent study has revealed that ANKRD1 expression decreased the sensitivity of ovarian cancer to drugs [14, 17]. We therefore treated SPC-A1 cells with different concentrations of carboplatin (CBP) and found that the IC_50_ values of the ANKRD29 overexpressed group were significantly lower than control group (Fig. 5G), indicating that ANKRD29 might regulate the drug sensitivity of NSCLC cells to CBP. Moreover, as mentioned above, ANKRD29 overexpression inhibited cell growth and migration ability of H1975 (a lowly sensitive cell line in Novartis DRIVE database, Fig. S3B), we established stable ANKRD29-overexpressed cell lines in H1299 (a highly sensitive cell line in Novartis DRIVE database, Fig. S3A) and found that increased expression of ANKRD29 also restrained cell proliferation and migration of H1299 compared to indicated control group through colony formation and wound healing assays (Fig. S3K-O). These data above supported the tumor suppressor role of ANKRD29 in NSCLC.

### ANKRD29 was involved in the NSCLC tumor microenvironment (TME) regulation and predicted the response to cancer immunotherapy

**Fig. 6:**
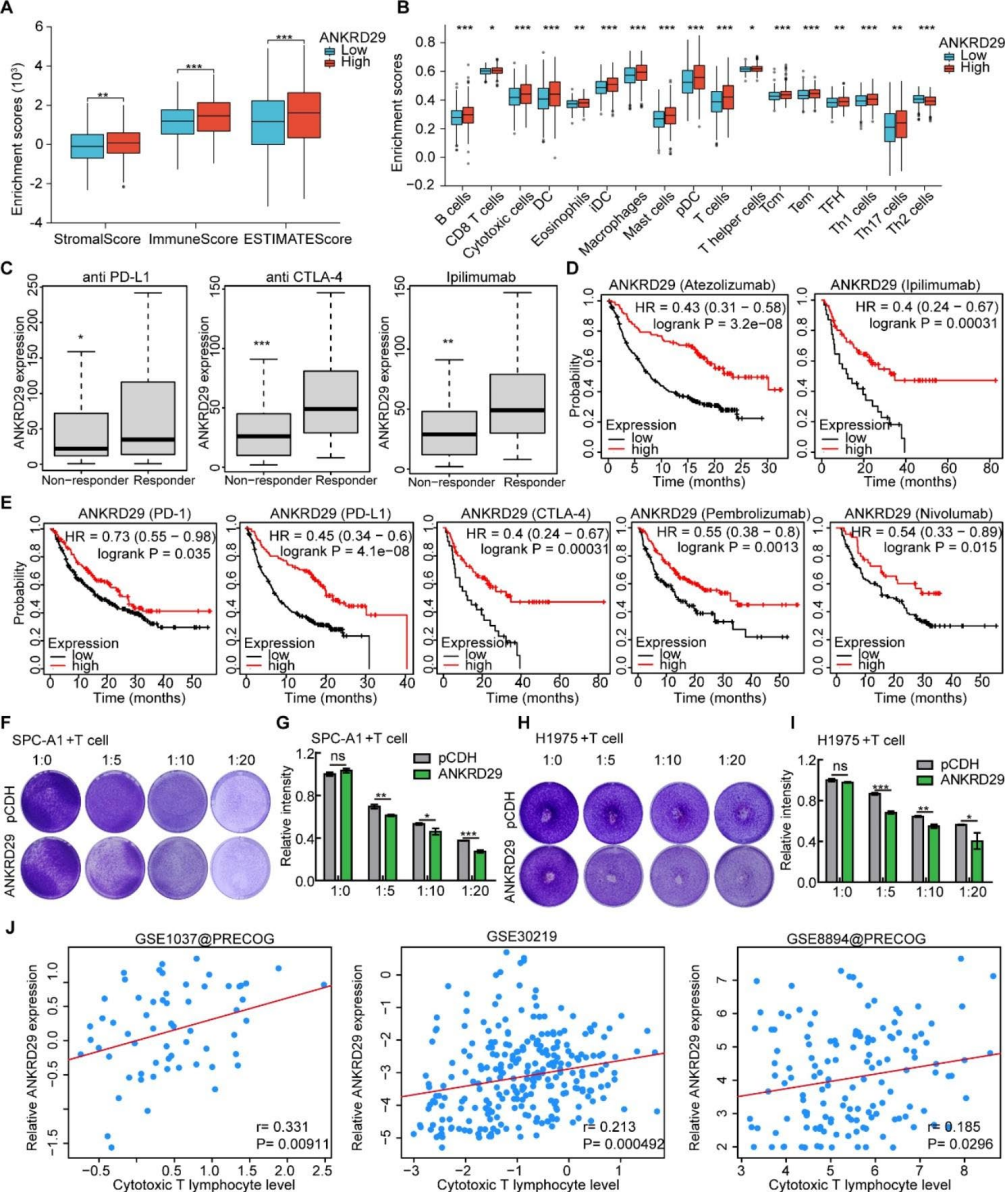
Correlation analysis of ANKRD29 expression on immune cell infiltration and immunotherapy in NSCLC. **A-B** Three immune-related scores (ESTIMATE score, stromal score and immune score) (**A**) and immune cell enrichment scores (**B**) showed that ANKRD29 expression was positively correlated with the level of immune cell infiltration levels. **C-E** Data obtained from KM plotter website showed that patients with low expression of ANKRD29 indicated promising responses to anti-PD1, anti-CTLA-4 and PD-L1/Atezolizumab therapy (**D-E**), in line with the increased expression of ANKRD29 in ICB responders analyzed by ROC Plotter database (**C**). **F-I** Overexpression of ANKRD29 in cancerous cells enhanced the ability of Jurkat to kill SPC-A1 and H1975 cells. Representative crystal violet staining images of SPC-A1 and H1975 cells after co-culture with Jurkat cells (**F**, **H**). (**G**, **I**) Quantification data for (**F**, **H**), respectively. **J** Correlation analysis between ANKRD29 expression and cytotoxic T lymphocyte infiltration levels in NSCLC datasets from TIDE website. Bars are the mean value ± SD. ns = no significant. * P < 0.05, ** P < 0.01, *** P < 0.001

As documented previously, tumor microenvironment (TME) played pivotal roles in NSCLC tumorigenesis and drug resistance [36, 37], we therefore examined whether ANKRD29 was involved in the NSCLC TME regulation. Surprisingly, we revealed that ANKRD29 expression notably correlated negatively with immune cells infiltration level using ESTIMATE algorithm analysis (a scoring method for tumor purity where the level of stromal cells and the infiltration level of immune cells in tumor tissues based on expression data present), which was consistent with the results that high ANKRD29 expression was positively related with different immune cells infiltration level by Single-sample gene set enrichment analysis (ssGSEA) in NSCLC (Fig. 6A, B). Additionally, we examined the potential therapeutic effects of ANKRD29 upon the predicted therapeutic efficacy and patient outcome of immune checkpoint blockers (ICBs) sub-cohort. The area under the curve (AUC) score of ANKRD29 for predicting ICB effects of NSCLC was 0.7, which was better than TIDE, MSI and IFNG scores prediction efficiency and similar with CD274, CD8 and Merck18 scores (Fig. S4A) [38]. We also discovered that copy numbers variations (CNVs) of ANKRD29 could affect the infiltration of B cells, CD8 + T cells, CD4 + T cells, macrophages, neutrophils and dendritic cells in LUAD, and B cells, CD4 + T cells, macrophages, neutrophils and dendritic cells in LUSC extracted from the TIMER database (Fig. S4B). Considering the different ANKRD29 expression in immune subtypes of LUAD and LUSC, we analyzed the expression of ANKRD29 in different cell types of NSCLC from TISCH2, a published single-cell RNA sequencing dataset [31], and found that ANKRD29 predominantly expressed in malignant tumor cells (Fig. S4C, D).

A recent study has revealed that the immunophenoscore (IPS) score could be served as a marker to predicate the immunogenicity of cancers and intratumoral immunologic landscape [32]. Therefore, we examined the relationship between ANKRD29 expression and IPS scores in pan-cancers. As expected, ANKRD29 expression was associated with IPS scores including MHC-related molecules (MHC), checkpoints or immunomodulators (CP), effector cells (EC), suppressor cells (SC) and average Z-score (AZ) in multiple tumors (Fig. 6F). Furthermore, ANKRD29 expression was positively related with most immunostimulators, MHC molecules and chemokines expression in LUAD and LUSC from TISIDB website [39] (Fig. S4E, F), which suggesting that ANKRD29 expression was involved in cancer cell immune infiltration level. Meanwhile, patients with high expression of ANKRD29 represented promising responses to anti-PD-1, anti-CTLA-4 and anti-PD-L1 therapy extracted from ROC Plotter and KM plotter websites (Fig. 6C-E) [40, 41]. Moreover, we revealed that overexpression of ANKRD29 in NSCLC cells promoted the ability of Jurkat cells to kill SPC-A1 and H1975 cells (Fig. 6F-I). Consistently, ANKRD29 expression was positively correlated with cytotoxic T lymphocyte level from TIDE database. Altogether, these data showed that ANKRD29 may also played important roles in NSCLC ICBs response (Fig. 6J).

### ANKRD29 was a potential target for NSCLC therapy

**Fig. 7:**
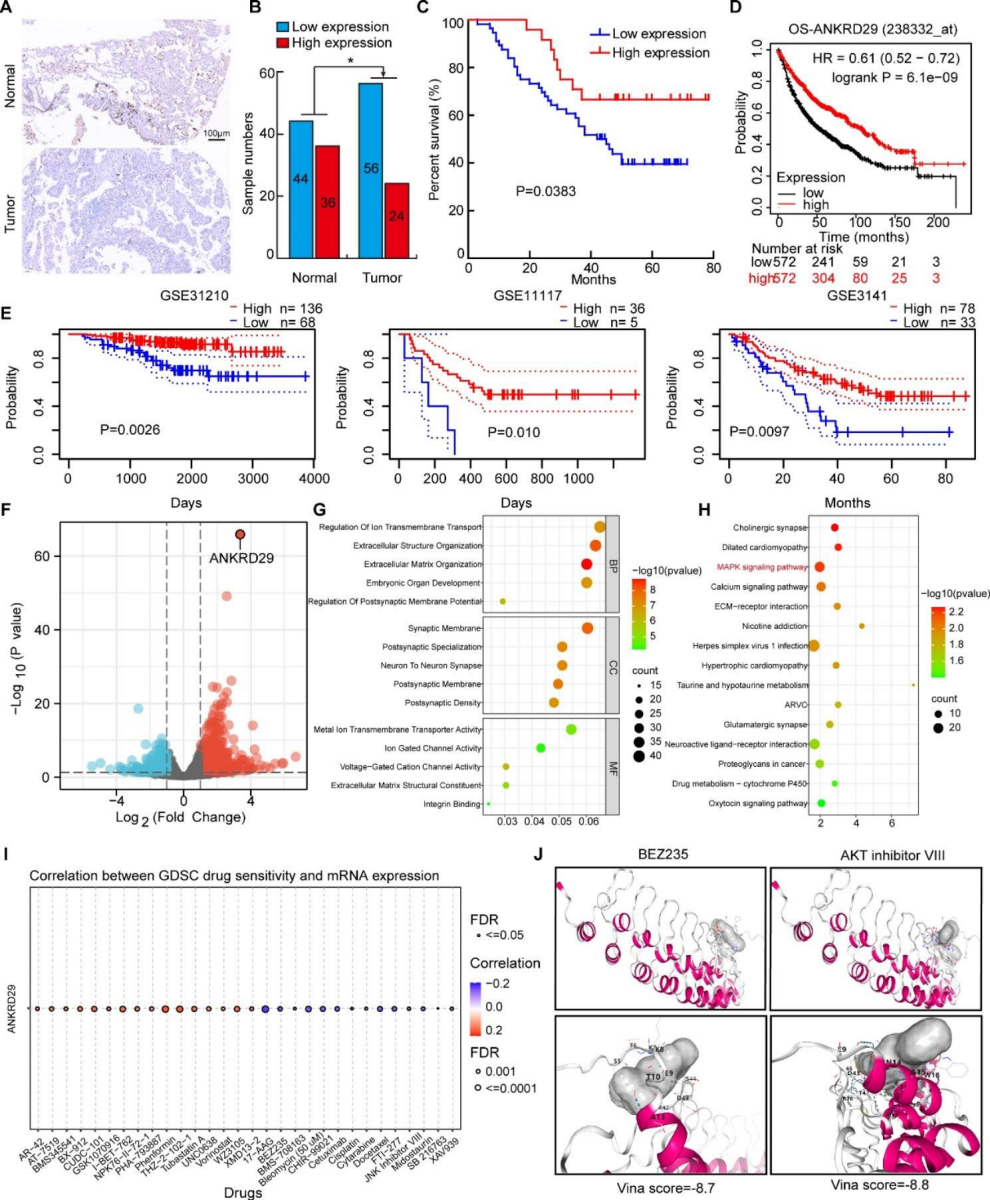
ANKRD29 was a potential target for NSCLC treatment. **A-B** Representative IHC images of ANKRD29 in NSCLC tissue microarray. (**B**) Quantification data for (**A**), Scale bar = 100 μm. **C-E** NSCLC patients with low ANKRD29 expression had a worse prognosis examined by tissue microarray (**C**), KM plotter (**D**) and PrognoScan websites (**E**), respectively. **F** Volcano plot showed differentially expressed genes (DEGs) after overexpression of ANKRD29 expression by using RNA-seq analysis in H1975. **G-H** GO (**G**) and KEGG (**H**) analysis using DEGs enriched the pathways affected by upregulation of ANKRD29 expression. **I** Pearson correlation analysis was performed between ANKRD29 expression and drug IC_50_ using the GDSC database. **J** Molecular docking profiles of BEZ235-2 and AKT inhibitor VIII with ANKRD29. Bars are the mean value ± SD. ns = no significant. * P < 0.05, ** P < 0.01, *** P < 0.001

To validate the expression level of ANKRD29 in clinical NSCLC samples, the immunohistochemistry (IHC) assay was applied in NSCLC tissue microarrays (TMA) containing 80 NSCLC tumor tissues and paired adjacent normal tissues. We found that the expression level of ANKRD29 was significantly decreased in tumor tissues compared to normal tissues in TMA (Fig. 7A, B; Table S6). And consistent with our findings in the TCGA-NSCLC cohorts, NSCLC patients with lower ANKRD29 expression exhibited worse OS time in TMA data, KM plotter and PrognoScan websites (Fig. 7C-E) [41, 42]. To decipher the underlying mechanism of ANKRD29 in NSCLC progression, RNA-seq analysis was performed to cramp out downstream signaling pathways mediated by ANKRD29. We identified 678 up-regulated and 286 down-regulated genes affected by ANKRD29 overexpression in H1975 cells (|log2(FC)| > 1 and P < 0.05) (Fig. 7F). Additionally, Gene Ontology (GO) enrichment and Kyoto Encyclopedia of Genes and Genomes (KEGG) pathway analysis showed that ANKRD29 may inhibited NSCLC tumorigenesis through regulating MAPK signaling pathway (Fig. 7G, H). In order to evaluate the clinical application potential of ANKRD29, we screened the correlation between ANKRD29 expression and drug sensitivity in pan-cancer from GDSC database (Fig. 7I). At last, ANKRD29 protein may directly interact with BEZ235 and AKT inhibitors VIII through molecular dock assays, which suggesting BEZ235 and AKT inhibitors VIII were potential agonists for ANKRD29 activation in CB-dock websites (The lower of the vina score was, the interaction capacity was promising.). (Fig. 7J) [43, 44].

## Discussion

In the past decades, many oncogenes and tumor suppressor genes have been identified and used in targeted therapy for NSCLC, which has improved the survival and quality of life in several patients [45–47]. Unfortunately, the mechanisms underlying the pathogenesis and progression of NSCLC remains unclear. For most patients, surgery, radiotherapy and chemotherapy are still their main treatment options due to the lack of effective biomarkers [48, 49]. Therefore, it is particularly important to develop new molecular targets for NSCLC therapy.

Ankyrin repeat domain is one of the most widespread protein structural motifs in nature, usually with 33–34 amino acid residues, which form a helix-turn-helix structure depending on the mutual folding of structural regions and functions as mediators to regulate protein-protein interactions [50, 51]. ANK repeat proteins are involved in many processes, including the formation of transcription complexes, the initiation of immune responses, the biogenesis and assembly of cation channels in membranes, and the regulation of the cell cycle [8]. Several studies have identified a number of ankyrin repeat proteins that can form functional complexes by binding to corresponding targets, thereby promoting or inhibiting tumorigenesis and progression. For example, the p16 protein is a member of the INK-4 protein family, which contains four ANK repeat sequences in its structure. The α-helix face of its ANK structural domain and the intermediate β-hairpin together bind the C-termin and N-termin of CDK6, locking the kinase in a conformation that is not conducive to activation, thereby regulating the cell cycle and exerting a tumor suppressor function [52–54]. Higashitsuji H et al. identified Gankyrin, an oncogenic protein that contained six ANK sequences in hepatocellular carcinoma, which promoted phosphorylation and degradation of Rb proteins by binding Rb proteins and cyclin-dependent kinases. Gankyrin could also promote ubiquitination and degradation of p53 by binding to P53 and interacting with MDM2, a negative regulator of p53 [55–57].

ANKRD29, located at chromosome 18q11.2, is an ankyrin repeat domain protein consisting of eight repeats of the ANK repeat. Up to now, there is limited information related to ANKRD29 in cancer progression including NSCLC. In this study, we firstly constructed a risk-score system for predicting prognosis of NSCLC patients and identified 5 hub ANKRDs including ANKRD29 by integrative bioinformatical analysis. Moreover, we revealed that ANKRD29 was lowly expressed in cancerous tissues compared to normal tissues, and its low expression in NSCLC patients predicted a poor prognosis. Meanwhile, promoter methylation was the cause of ANKRD29 low expression in NSCLC. Then we constructed ANKRD29 overexpression cell lines and found that forced-expression of ANKRD29 inhibited NSCLC cells’ proliferation, migration and induced cell cycle arrest at G0/G1 phase. In addition, we proved that ANKRD29 played an important role in enhancing drug sensitivity and regulating immune cell infiltration. RNA-seq results showed that ANKRD29 participated in NSCLC tumorigenesis through regulating MAPK signaling pathway. And importantly, we screened BEZ235 and AKT inhibitors VIII were potential agonists for ANKRD29 activation. The above evidence suggested that ANKRD29 functioned as tumor suppressor in NSCLC.

In conclusion, we identified a new tumor suppressor gene, ANKRD29, which is commonly down-regulated in NSCLC tumor tissues. ANKRD29 exerted tumor suppressive effects in NSCLC by regulating tumor cell proliferation, migration, EMT, drug sensitivity and immune infiltration. Our results suggested that ANKRD29 is a valuable biomarker and molecular target for NSCLC treatment in the future.

## Conclusions

We found that the ANKRD29 expression was decreased in NSCLC and revealed that high ANKRD29 expression obviously correlated with patients’ better clinical outcome. Overexpression of ANKRD29 significantly inhibited NSCLC cells’ proliferation and migration, and promoted the cancerous cells’ sensitivity to carboplatin and enhanced the killing ability of T cells in NSCLC cells. RNA-seq results showed that ANKRD29 could regulate MAPK signaling pathway. Moreover, we screened two potential agonists for ANKRD29. Therefore, ANKRD29 might be a diagnostic marker and therapeutic target of NSCLC.

## Electronic supplementary material

Below is the link to the electronic supplementary material.

Supplementary Material 1

Supplementary Material 2

Supplementary Material 3

Supplementary Material 4

## Data Availability

Datasets analyzed in this study can be obtained from the corresponding author on reasonable request. The datasets presented in this study can be found in online repositories. The names of the repository/repositories and accession number(s) can be found in the article/supplementary material.

## Abbreviations

5-Aza: 5-Azacytidine
ANKRD: ankyrin repeat domain
AUC: area under the curve
ATCC: American type culture collection
AZ: average Z-score
CBP: carboplatin
CNV: copy numbers variation
CP: checkpoints
DEG: differential expression gene
EC: effector cell
EMT: epithelial mesenchymal transition
FBS: fetal bovine serum
GEO: Gene Expression Omnibus
GO: Gene Ontology
GSCA: Gene Set Cancer Analysis
ICB: immune checkpoint blocker
IHC: Immunohistochemistry
IPS: immunophenoscore
KEGG: Kyoto Encyclopedia of Genes and Genomes
KM: Kaplan-Meier
LUAD: lung adenocarcinoma
LUSC: lung squamous carcinoma
NCBI: National Center for Biotechnology Information
NSCLC: non-small cell lung cancer
OS: overall survival
PPI: protein-protein interaction
ROC: receiver operating characteristic
RSS: Risk Score System
SC: suppressor cell
SRB: sulforhodamine B
TCGA: The Cancer Genome Atlas
TMA: tissue microarrays
TME: tumor microenvironment
WB: western blot
