# Supplementary material for: ANKRD29, as a new prognostic and immunological biomarker of non–small cell lung cancer, inhibits cell growth and migration by regulating MAPK signaling pathway
Source: Biol Direct. 2023 Jun 6;18:28. doi: 10.1186/s13062-023-00385-7 (PMC10243072; doi:10.1186/s13062-023-00385-7)
Supplement: Supplementary file 1 — Supplementary Material 1 [file 13062_2023_385_MOESM1_ESM.pdf]

ANKRD29, as a new prognostic and immunological biomarker of non–small cell lung cancer, inhibits cell growth and migration by regulating MAPK signaling pathway

Hanqing Zhao [1, †], Yanbo Wang [1,2, †], Yaomei He [4,5,6, †], Peng Zhang [1,2], Cheng Zeng [1], Tongxuan Du [3], Qiushuo Shen [1,3, *], Song Zhao [1, *]

1. Department of Thoracic Surgery, the First Affiliated Hospital of Zhengzhou University, Zhengzhou 450052, China.
2. Academy of Medical Science, Zhengzhou University, Zhengzhou 450052, China.
3. Institute of Biomedical Engineering, Kunming Medical University, Kunming, Yunnan 650500, China.
4. Yan'an Hospital Affiliated to Kunming Medical University, Kunming, China.
5. Key Laboratory of Tumor Immunological Prevention and Treatment in Yunnan Province, China.
6. Center for Life Sciences, School of Life Sciences, Yunnan University, Kunming, Yunnan, China.

† Hanqing Zhao, Yanbo Wang and Yaomei He contributed equally to this work.

*Correspondence: shenqiushuo@126.com; zhaosong@zzu.edu.cn.
