# Supplementary material for: ANKRD29, as a new prognostic and immunological biomarker of non–small cell lung cancer, inhibits cell growth and migration by regulating MAPK signaling pathway
Source: Biol Direct. 2023 Jun 6;18:28. doi: 10.1186/s13062-023-00385-7 (PMC10243072; doi:10.1186/s13062-023-00385-7)
Supplement: Supplementary file 2 — Supplementary Material 2 [file 13062_2023_385_MOESM2_ESM.docx]

**Supplementary Materials**

**Supplementary Fig. S1**


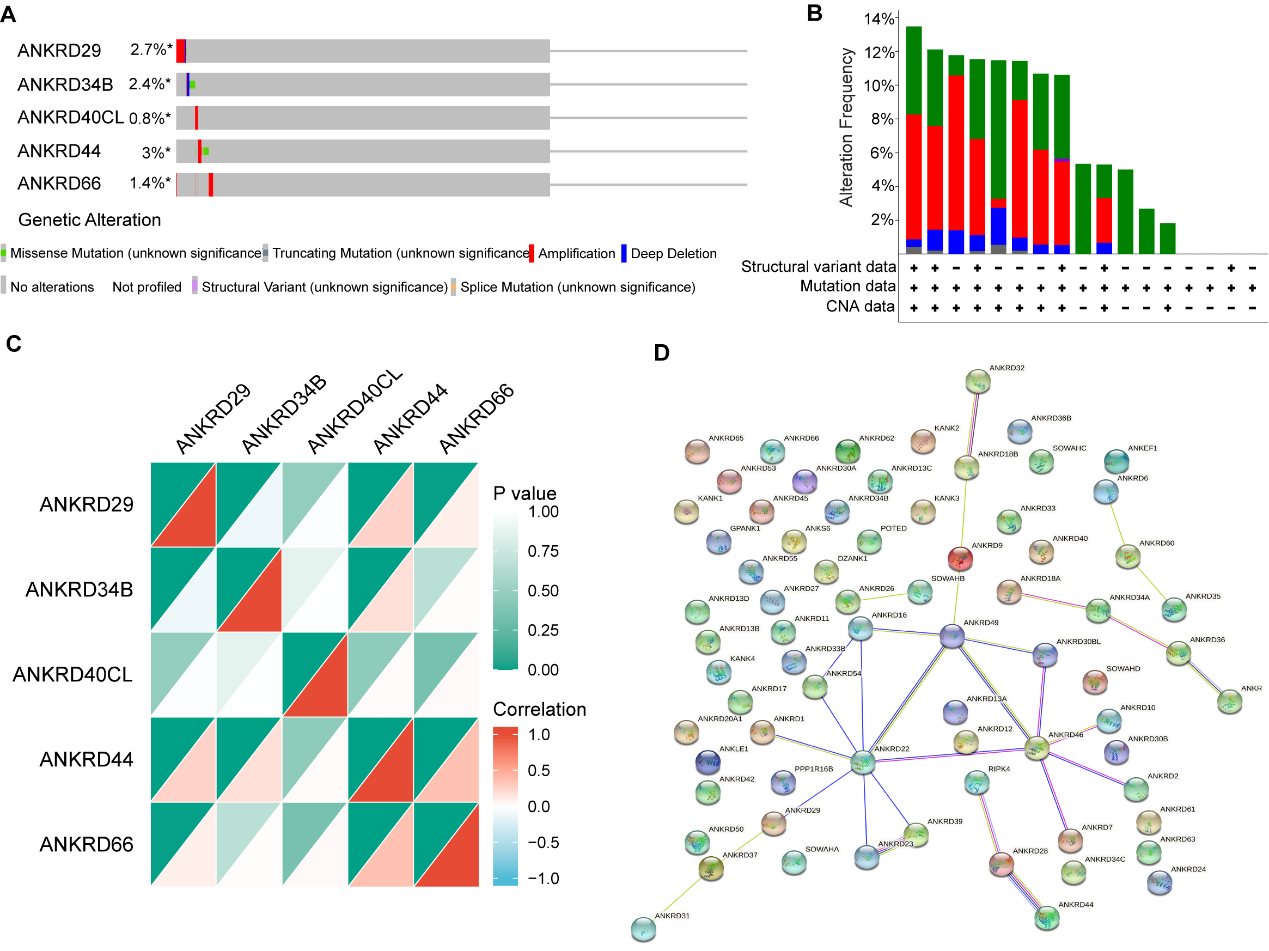


**Fig. S1** Mutation and interaction analysis of ANKRD genes. **A** Mutation status of five ANKRD genes in NSCLC patients from cBioPortal database. **B** Histogram of genetic mutation frequency of the five ANKRD genes in NSCLC patients from cBioPortal database. **C** Co-expression heatmap of five ANKRD genes in NSCLC. **D** Protein-protein interaction network of 75 ANKRD proteins. Bars are the mean value ± SD. ns=no significant. * P < 0.05, ** P < 0.01, *** P < 0.001.

**Supplementary Fig. S2**


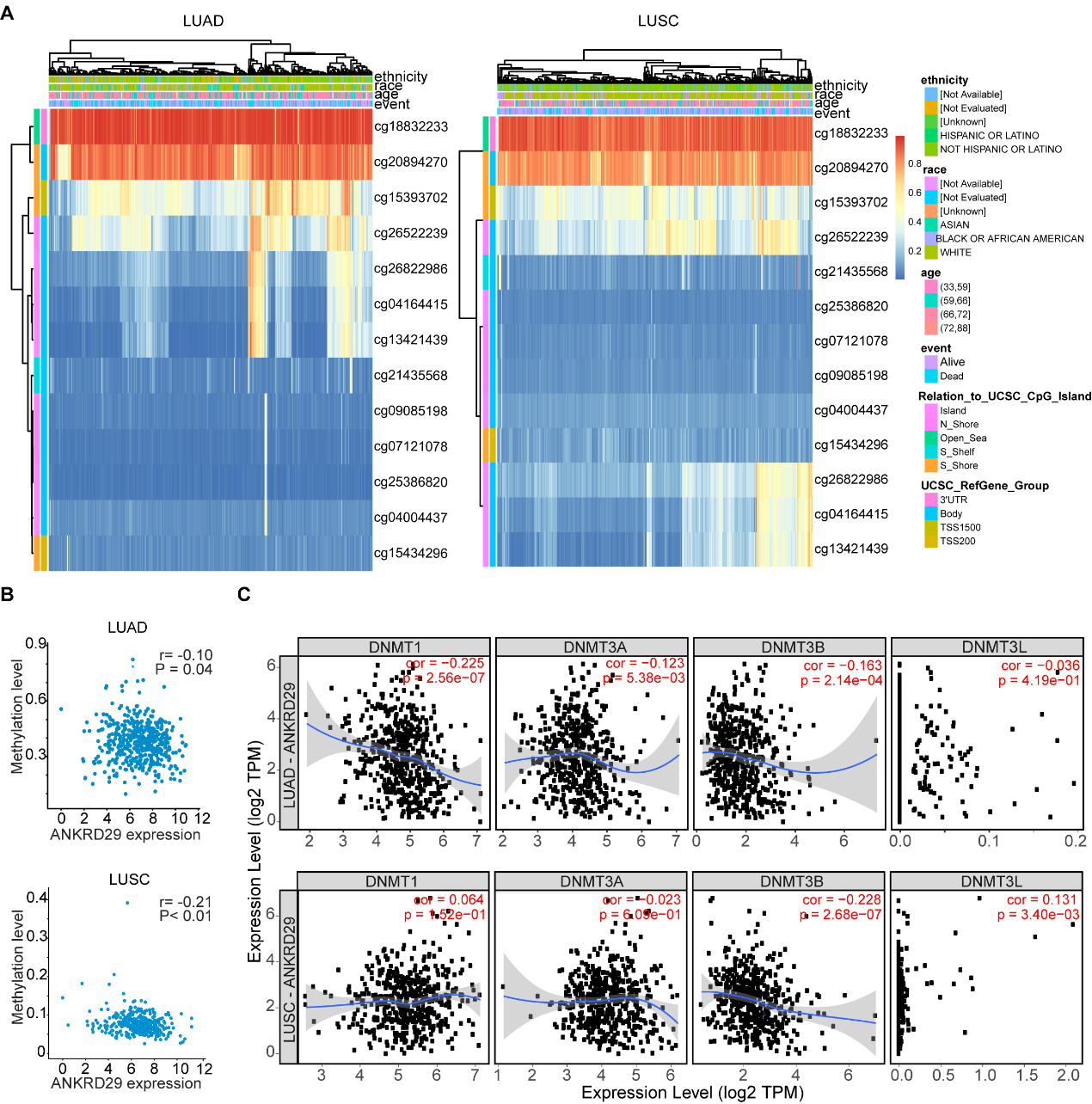


**Fig. S2** Methylation analysis of ANKRD29 promoter in NSCLC from TCGA data. **A** Heat map to visualize ANKRD29 expression levels and methylation in LUAD and LUSC. **B** Correlation of ANKRD29 expression levels with methylation in LUAD and LUSC analyzed by cBioPortal database. **C** Correlation analysis between ANKRD29 expression and DNMT1, DNMT3A, DNMT3B and DNMT3L from the TIMER website.


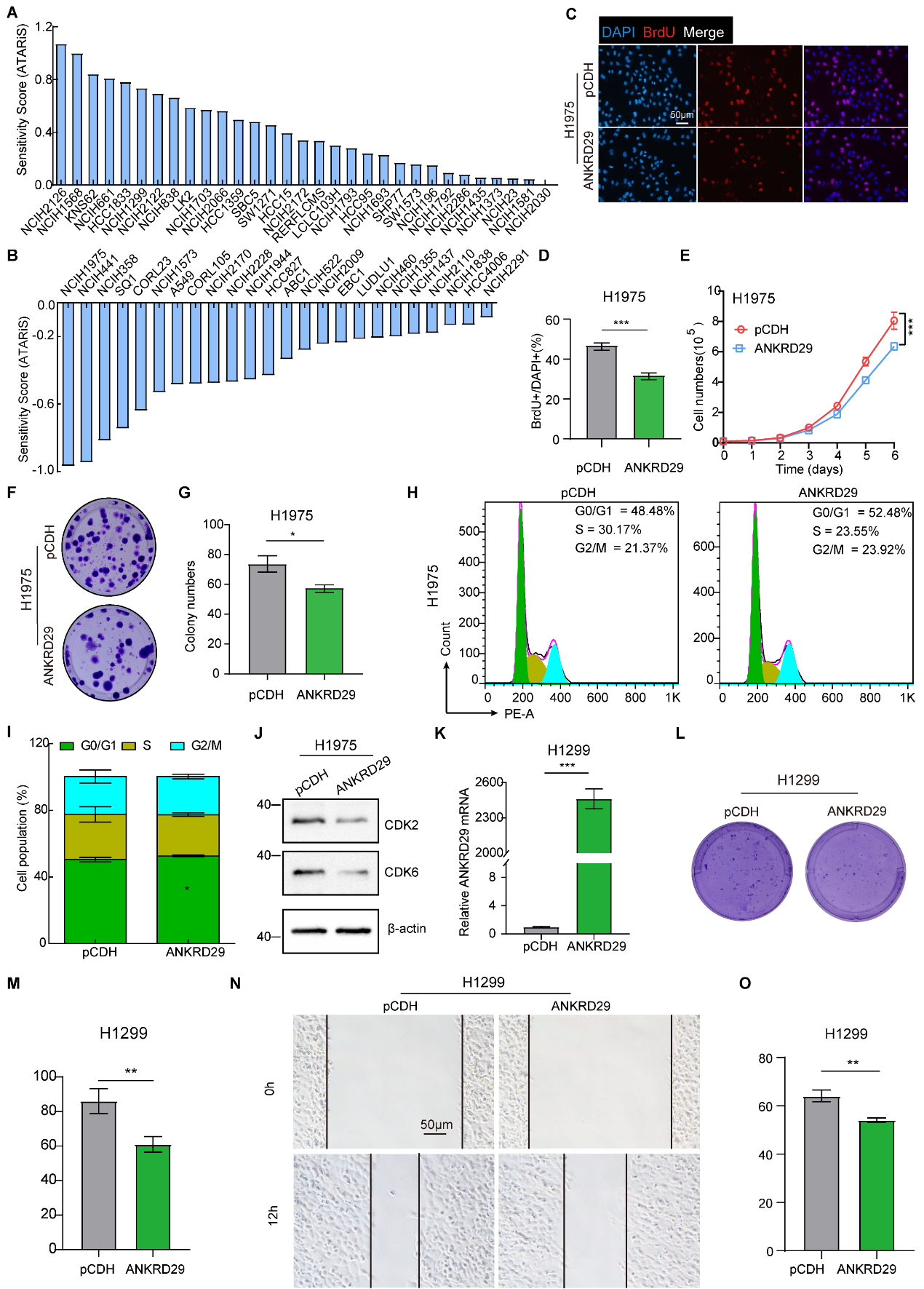


**Fig. S3** Overexpression of ANKRD29 inhibited NSCLC cells’ proliferation and migration ability. **A-B** ATARiS profiles of ANKRD29 shRNA in various NSCLC cell lines shRNA library database (https://oncologynibr.shinyapps.io/drive/). **C-G** Overexpression of ANKRD29 obviously inhibited H1975 cell growth through BrdU incorporation abilities (**C-D**), growth curve (**E**) and colony formation (**F-G**). (**D**) and (**G**) Quantification data for (**C**) and (**F**), respectively. **H-I** Overexpression of ANKRD29 induced cell cycle arrest at G0/G1 phase analyzed by flow cytometry assay. (**I**) Quantification data for (**H**). **J** ANKRD29 elevated expression downregulated the protein level of CDK2 and CDK6. **K** qRT-PCR assays were used to verify the overexpression of ANKRD29 in H1299 cell lines. **L**-**O** Overexpression of ANKRD29 inhibited H1299 cell growth and migration through colony formation (**L-M**) and wound-healing assays (**N-O**). (**M**) and (**O**) Quantification data for (**L**) and (**N**), respectively. Scale bar=50 μm. Bars are the mean value ± SD. ns=no significant. * P < 0.05, ** P < 0.01, *** P < 0.001.


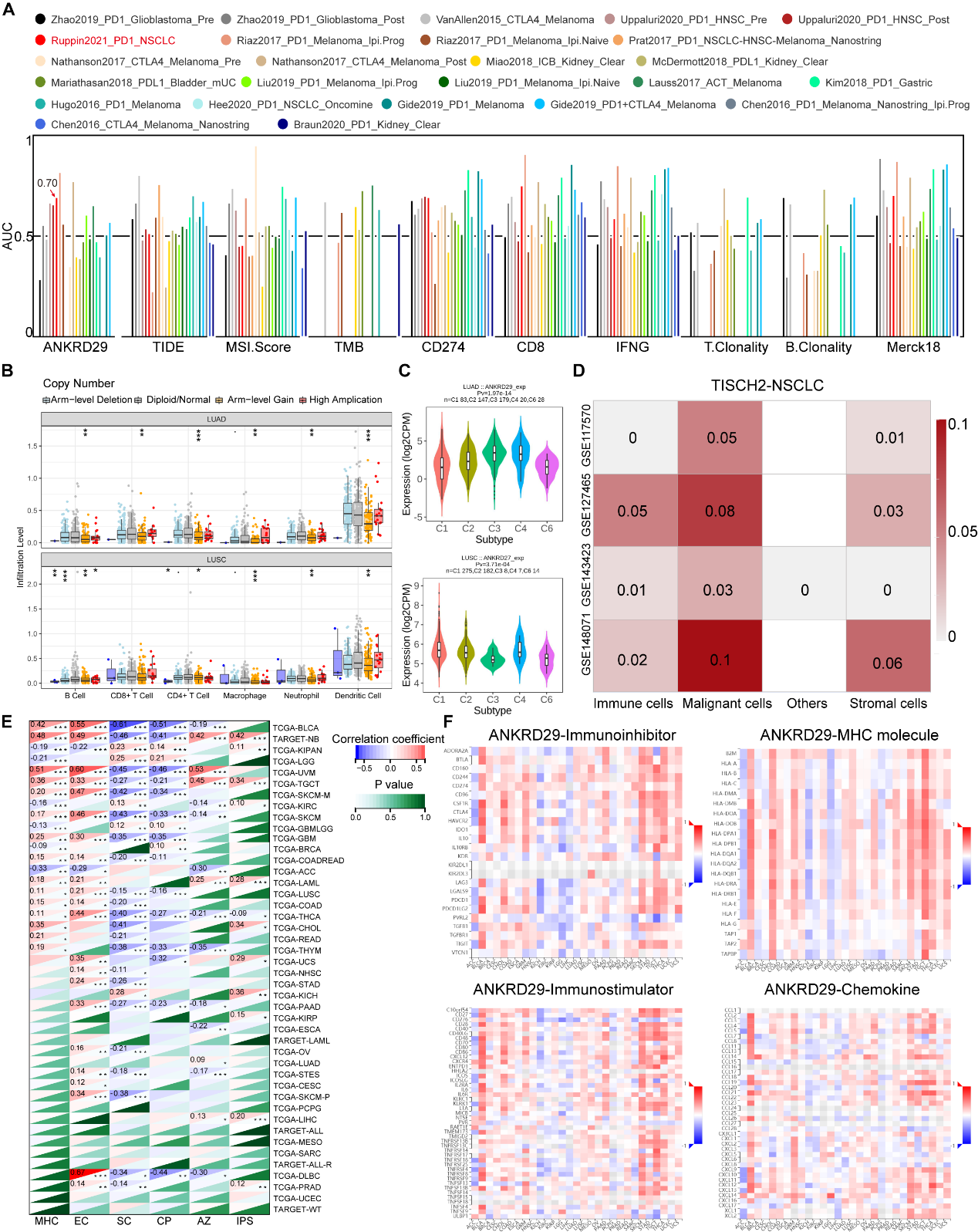


**Fig. S4** Correlation analysis between ANKRD29 expression and tumor infiltration levels of NSCLC. **A** The bar chart showing the difference in the efficacy of ANKRD29 and standardized cancer immune evasion biomarkers in various data sets from TIDE website. **B** Correlation between cell copy number variations and infiltration levels of 6 types of immune cells in LUAD and LUSC from TIMER database. **C** Analysis of ANKRD29 expression levels and expression of different immunological subtypes in LUAD and LUSC. C1: wound healing; C2: IFN-gamma dominant; C3: inflammatory; C4: lymphocyte depleted; C6: TGF-β dominant. **D** Heat map showed the expression profile of ANKRD29 in NSCLC extracted from TISCH2, a published single-cell RNA sequencing dataset (31). **E** The correlation between ANKRD29 expression level with immunophenoscore (IPS) including MHC-related molecules (MHC), checkpoints or immunomodulators (CP), effector cells (EC), suppressor cells (SC) and average Z-score (AZ) in pan-cancer. **F** Heatmap analysis of ANKRD29 expression and immunostimulantors, immunoinhibitors. ns=no significant. * P < 0.05, ** P < 0.01, *** P < 0.001.

**Table S1. ANKRDs selected in this study (see attached Excel file named Supplementary Materials 2).**

**Table S2. Overlapping genes between TCGA-NSCLC DEGs and ANKRDs (see attached Excel file named Supplementary Materials 2).**

**Table S3. Univariate analysis of 21 ANKRDs in NSCLC (see attached Excel file named Supplementary Materials 2).**

**Table S4. The coefficients of five risk factors by multivariate analysis in NSCLC (see attached Excel file named Supplementary Materials 2).**

**Table S5. Univariate and multivariate Cox analysis of OS in TCGA-NSCLC dataset (see attached Excel file named Supplementary Materials 2).**

**Table S6. The pathological characteristics of patients with ANKRD29 (n=80) in tissue microarray.**

| Characteristic | levels | Overall |
| --- | --- | --- |
| n |  | 80 |
| Age, median (IQR) | | 60 (53, 67) |
| Gender, n (%) | Female | 50 (62.5%) |
|  | Male | 30 (37.5%) |
| T stage, n (%) | T1 | 28 (35%) |
|  | T2 | 43 (53.8%) |
|  | T3 | 4 (5%) |
|  | T4 | 5 (6.2%) |
| N stage, n (%) | N0 | 53 (66.2%) |
|  | N1 | 10 (12.5%) |
|  | N2 | 17 (21.2%) |
| M stage, n (%) | M0 | 71 (88.8%) |
|  | M1 | 9 (11.2%) |
| Pathologic stage, n (%) | Stage I | 22 (27.5%) |
|  | Stage II | 21 (26.2%) |
|  | Stage III | 28 (35%) |
|  | Stage IV | 9 (11.2%) |
| Survival status, n (%) | Alive | 39 (48.8%) |
|  | Death | 41 (51.2%) |

**Table S7. The databases information used in this study.**

| **Name** | **Keywords** | **Link** | **Targets** |
| --- | --- | --- | --- |
| GSCA | Gene Set Cancer Analysis | http://bioinfo.life.hust.edu.cn/GSCA/#/expression | To examine the gene mutation pattern, methylation level and targeting drugs. |
| MethSurv | DNA methylation and its relation to cancer survival | https://biit.cs.ut.ee/methsurv/ | To examine the methylation status. |
| cBioportal |  | http://www.cbioportal.org/ | To examine the mutation pattern of ANKRD29 in NSCLC. |
| TCGA |  | https://portal.gdc.cancer.gov/ | To examine the expression of ANKRD29 and other gene in NSCLC. |
| UALCAN |  | http://ualcan.path.uab.edu/ | To examine the methylation status. |
| Novartis DRIVE |  | https://oncologynibr.shinyapps.io/drive/ | To examine the function of ANKRD29 in NSCLC cells. |
| KEGG | Kyoto Encyclopedia of Genes and Genomes | https://www.genome.jp/kegg/catalog/org_list.html | To examine ANKRD29 regulated MAPK signaling pathway. |
| GO | Gene Ontology | http://geneontology.org/ | To examine ANKRD29 regulated MAPK signaling pathway. |
| TIMER |  | http://timer.cistrome.org/ | To examine the correlation between ANKRD29 and immune infiltration. |
| Kaplan_Meier Plotter |  | https://kmplot.com/analysis/index.php?p=background | To examine the correlation between ANKRD29 expression and survival time of NSCLC patients. |
| PrognoScan |  | http://dna00.bio.kyutech.ac.jp/PrognoScan/index.html | To examine the correlation between ANKRD29 expression and survival time of NSCLC patients. |
| ROC Plotter |  | https://www.rocplot.org/ | To examine the correlation between ANKRD29 expression and prognosis of NSCLC patients. |
| CB-Dock |  | http://clab.labshare.cn/cb_dock/php/index.php | To verify the candidate drugs. |
| TIDE |  | http://tide.dfci.harvard.edu/ | To determine the TIDE score of NSCLC patients using TCGA datasets. |
| TISIDB |  | http://cis.hku.hk/TISIDB/ | To determine the correlation between ANKRD29 expression in different immunological subtypes of NSCLC. |
| SMART |  | http://www.bioinfo-zs.com/smartapp/ | To determine the methylation level of ANKRD29 promoter in NSCLC. |
| TISCH2 |  | http://tisch.comp-genomics.org/ | To determine the expression of ANKRD29 in different cell types of NSCLC. |

**Table S8. Antibodies and oligos used in this study.**

| **Antibody Name** | **Catalog number** | **Supplier** | **Species** |
| --- | --- | --- | --- |
| β-actin | 60008-1-1g | Proteintech | Mouse |
| CDK2 | 10122 | Proteintech | Rabbit |
| CDK6 | 164821 | abcam | Rabbit |
| Snail | 3879S | CST | Rabbit |
| Slug | 9585S | CST | Rabbit |
| ANKRD29 (WB) | NBP2-33564 | Nouvs | Rabbit |
| ANKRD29 (IHC) | 23999-1-AP | Proteintech | Rabbit |
| BrdU | 66241 | Proteintech | Mouse |
| **qRT-PCR primers used in this work** | | | |
| ANKRD29 | Forward | GTGAGGGAACTGGTTCTGCAA | |
| ANKRD29 | Reverse | TGGTCCTAAATTCAGTGGATGC | |
| β-actin | Forward | AAGTGTGACGTGGACATCCGC | |
| β-actin | Reverse | CCGGACTCGTCATACTCCTGCT | |

**Table S9.** **The full names of cancer types were shown in this study.**

| **Cancer Type** | **Full Name** |
| --- | --- |
| BLCA | Bladder Urothelial Carcinoma |
| NB | Neuroblastoma |
| BRCA | Breast invasive carcinoma |
| CESC | Cervical squamous cell carcinoma and endocervical adenocarcinoma |
| COAD | Colon adenocarcinoma |
| ESCA | Esophageal carcinoma |
| KIRC | Kidney renal clear cell carcinoma |
| KIRP | Kidney renal papillary cell carcinoma |
| LIHC | Liver hepatocellular carcinoma |
| LUAD | Lung adenocarcinoma |
| LUSC | Lung squamous cell carcinoma |
| OV | Ovarian serous cystadenocarcinoma |
| PAAD | Pancreatic adenocarcinoma |
| PRAD | Prostate adenocarcinoma |
| READ | Rectum adenocarcinoma |
| SARC | Sarcoma |
| SKCM | Skin Cutaneous Melanoma |
| SKCM-P | Skin Cutaneous Melanoma-Primary |
| SKCM-M | Skin Cutaneous Melanoma-Metastasis |
| TGCT | Testicular Germ Cell Tumors |
| THCA | Thyroid carcinoma |
| THYM | Thymoma |
| UCEC | Uterine Corpus Endometrial Carcinoma |
| UCS | Uterine Carcinosarcoma |
| GBM | Glioblastoma multiforme |
| GBMLGG | Lower grade glioma and Glioblastoma |
| LGG | Lower Grade Glioma |
| COADREAD | Colon and Rectal Cancer |
| STAD | Stomach Cancer |
| HNSC | Head and Neck Cancer |
| ALL | Acute Lymphoblastic Leukemia |
| ALL-R | Relapsed or Refractory acute lymphoblastic leukemia |
| LAML | Acute Myeloid Leukemia |
| PCPG | Pheochromocytoma & Paraganglioma |
| ACC | Adrenocortical Cancer |
| KICH | Kidney Chromophobe |
| CHOL | Bile Duct Cancer |
| MESO | Mesothelioma |
| DLBC | Large B-cell Lymphoma |
| WT | Wilms Tumor |
| UVM | Uveal Melanoma |
| KIPAN | Pan-kidney cohort (KICH+KIRC+KIRP) |
| STES | Stomach and Esophageal carcinoma |
| PCPG | Pheochromocytoma and Paraganglioma |

**Table** **S10. The correlation between ANKRD29 expression and GDSC drugs sensitivity in pan-cancer (see attached Excel file named Supplementary Materials 3).**
